# Genetic evolution of low pathogenecity H9N2 Avian influenza viruses in Tunisia: acquisition of new mutations

**DOI:** 10.1186/1743-422X-8-467

**Published:** 2011-10-12

**Authors:** Wafa Tombari, Jihene Nsiri, Imen Larbi, Jean Luc Guerin, Abdeljelil Ghram

**Affiliations:** 1 Veterinary Microbiology laboratory, Pasteur Institute of Tunis, 1002 Tunis-Belvédère, Tunisia; 2 INRA, UMR 1225, Ecole nationale vétérinaire de Toulouse, F-31076 Toulouse, France

## Abstract

**Background:**

Since the end of 2009, H9N2 has emerged in Tunisia causing several epidemics in poultry industry resulting in major economic losses. To monitor variations of Influenza viruses during the outbreaks, Tunisian H9N2 virus isolates were identified and genetically characterized.

**Methods:**

The genomic RNA segments of Tunisian H9N2 strains were subjected to RT-PCR amplifications followed by sequencing analysis.

**Results:**

Phylogenetic analysis demonstrated that A/Ck/TUN/12/10 and A/Migratory Bird/TUN/51/10 viruses represent multiple reassortant lineages, with genes coming from Middle East strains, and share the common ancestor Qa/HK/G1/97 isolate which has contributed internal genes of H5N1 virus circulating in Asia. Some of the internal genes seemed to have undergone broad reassortments with other influenza subtypes. Deduced amino acid sequences of the hemagglutinin (HA) gene showed the presence of additional glycosylation site and Leu at position 234 indicating to binding preference to α (2, 6) sialic acid receptors, indicating their potential to directly infect humans. The Hemagglutinin cleavage site motif sequence is ^333 ^PARSSR*GLF^341 ^which indicates the low pathogenicity nature of the Tunisian H9N2 strains and the potential to acquire the basic amino acids required for the highly pathogenic strains. Their neuraminidase protein (NA) carried substitutions in the hemadsorption (HB) site, similar to those of other avian H9N2 viruses from Asia, Middle Eastern and human pandemic H2N2 and H3N2 that bind to α -2, 6 -linked receptors. Two avian virus-like aa at positions 661 (A) and 702 (K), similar to H5N1 strains, were identified in the polymerase (PB2) protein. Likewise, matrix (M) protein carried some substitutions which are linked with increasing replication in mammals. In addition, H9N2 strain recently circulating carried new polymorphism, "GSEV" PDZ ligand (PL) C-terminal motif in its non structural (NS) protein.

Two new aa substitutions (I) and (V), that haven't been previously reported, were identified in the polymerase and matrix proteins, respectively. Nucleoprotein and non-structural protein carried some substitutions similar to H5N1 strains.

**Conclusion:**

Considering these new mutations, the molecular basis of tropism, host responses and enhanced virulence will be defined and studied. Otherwise, Continuous monitoring of viral genetic changes throughout the year is warranted to monitor variations of Influenza viruses in the field.

## Background

Avian Influenza virus (AIV) is a member of the family *Orthomyxoviridae*; containing negative sense single stranded RNA [1]. Based on their pathogenecity, Two types of AIV have been described, namely a highly pathogenic type (HPAIV) that causes severe disease with high mortality, and low pathogenic type (LPAIV) inducing asymptomatic or mild infection [2]. AIV subtypes, namely H5, H7 and H9, currently endemic in poultry in some regions of the world, have been shown to be capable of infecting humans [3,4].

By 1997, H9N2 viruses have been isolated in multiple avian species including chickens, ducks, turkeys, quail, geese and pigeons, throughout Asia, the Middle East, Europe and Africa and for the first time from humans in Hong Kong and China, in 1999 [5-7]. They could emerge as human pathogens through reassortment in intermediate hosts, such as pigs [6] and in avian species, or through direct adaptation in human host [8]. These viruses produce significant disease problems in poultry resulting in great economic losses due to reduced egg production or high mortality with co-infection with other opportunistic pathogens such as infectious bronchitis viruses [9], *Staphylococcus aureus, Avibacterium paragallinarum*, *Escherichia coli*, or immune suppression [10].

Two distinct lineages of H9N2 influenza viruses have been defined, the North American lineage and the Eurasian lineages which consists of at least three sublineages represented by their prototype strains: A/quail/Hong Kong/G1/97 (G1-like), A/duck/HongKong/Y280/9 (Y280-like), A/Chicken/Beijing/1/94 (BJ94-like), and A/chicken/Korea/38349-P96323/96 (Korean-like) [11,12]. The A/quail/Hong Kong/G1/97 is thought to be the donor of 6 internal genes to the poultry and the human H5N1 viruses isolated in 1997 [8].

In addition, some of these currently circulating H9N2 strains have acquired human-like receptor specificity (α2, 6 sialic acids) [13] and there is concern about the potential risk for these viruses to cross the species barriers and affect human health as the consequences of genetic reassortments between mammalian and avian influenza virus.

Since the end of 2009, H9N2 has emerged in Tunisia causing several epidemics in poultry industry resulting in major economic losses. It should be noted that Tunisia was reportedly free from Avian Influenza infection [14].

In the present study, we report the isolation of two Tunisian H9N2 influenza viruses that were identified, and their genome sequences were analyzed. Phylogenetic analysis characterization was performed in comparison to H9N2 sequences available in the Genbank database.

## Materials and methods

Fecal and tissue samples were collected from affected commercial poultry and from migratory bird during the initial outbreak. H9N2 avian influenza viruses were isolated and identified using classical laboratory methods [15]. The isolates were propagated in specific-pathogen free (SPF) chicken embryonated eggs via the allontoic route, and the eggs were incubated for 72-96 h at 37°C. The collected allontoic fluids were subjected to hemagglutination and hemagglutination inhibition tests using H9 monospecific polyclonal antisera obtained from F.A.O. The genomic RNA segments of viruses were extracted using viral Trizol LS reagent (Invitrogen, Carlsbad, CA) according to the manufacturer's instruction. The first-strand cDNA was synthesized using SuperScript™ III Reverse transcriptase (Invitrogen, Carlsbad, CA) and appropriate upstream primers. cDNA was amplified by PCR using described IA specific primers listed in Table 1. 5 μl of cDNA was added to the PCR reaction mixture (25 μl) containing 2, 5 μl of 10× PCR buffer, 2, 5 μl of 2, 5 mM dNTPs, 0, 5 μl Taq DNA polymerase (5 units/μl, Invitrogen), 1 μl of each primer (10 pmol each), 2 μl of 5 Mm Mgcl_2 _(Invitrogen, Carlsbad, CA) and 17, 5 μl of H_2_O RNase free (BioBasic, Carlsbad, CA). The PCR program was 94^°^C for 2 min, 35 cycles of 94^°^C for 45 s, 50^°^C for 45 s [15-18] (58^°^C for HA1, NA, PB2, and NS) [19,20], 72^°^C for 1 min followed by 72^°^C for 10 min. PCR products were purified with gel DNA purification kit (GenClean^® ^II kit, North America, MP).

**Table 1:** Primer sequences as used in the RT-PCR in our study

Name	**Sequence (5'- 3')**^**a**^	Position	Expected products size (pb)	Reference
**AMF**	**CTTCTAACCGAGGTCGAAAC**	7- 26	244	15
**AMR**	**AGGGCATTTTGGACAAAKCGTCTA**	259-238		
**MF**	**CTCATGGAATGGCTAAAGACA**	149-169	700	16
**MR**	**CGATCAADAATCCACAATATC**	847-827		
**H9-1**	**CTYCACACAGARCACAATGG**	184-203	488	15
**H9-2**	**GTCACACTTGTTGTTGTRTC**	672-652		
**H9F**	**GAATCCAGATCTTTCCAGAC**	426-445	384	17
**H9R**	**CCATACCATGGGGCAATTAG**	808-789		
**NPF**	**CAGRTACTGGGCHATAAGRAC**	1200-1220	326	18
**NPR**	**GCA TTGTCTCCGAAGAAATAAG**	1529-1510		
**HAF1**	**GAATTGATTATTATTGGTCAGTA**	710-732	550	19
**HAR1**	**TCATCAATCT-TATTGTTGATCAT**	1272-1249		
**NAF**	**CTTGTTGGCGACACACCAAGRAA**	961-983	410	19
**NAR**	**GAGCCTGTTCCAT-AGGTACCTGA**	1370-1348		
**PB2F**	**TATTCAT-CRTCAATGATGTGGGA**	1591-1613	540	19
**PB2R**	**GATGCTYAATGCTGGTCCATATC**	2130-2108		
**NSF**	**AGCAAAAGCAGGGTGACAAA**	1-20	890	20
**NSR**	**AGTAGAAACAAGGGTGTTTT**	890-871		

^a ^Codes for mixed bases positions: D = G/A/T, H = A/C/T, K = G/T, R = A/G, Y = C/T.

ABI Big Dye Terminator version 1.1 sequencing Kit run on 3730 XL DNA Analyzed (ABI prism 377, DNA sequencer, Applied Biosystem Inc., CA, USA) sequencer was used for sequencing PCR products.

The Bioedit program 5.0.6 software and ClustalW alignement algorithm (version 1.83) were initially used to compare and align nucleotide sequences. Phylogenetic trees were constructed using MEGA5.01 program version 3.65 with neighbor-joining method. The robustness of the groupings in the neighbor-joining analysis was assessed with 1000 bootstrap resamplings. The Blast software and Bioedit programs were used to determine the sequence similarity of the Tunisian strains

**Nucleotide sequence accession numbers: **the nucleotide sequences obtained in this study were deposited in GenBank data library under accession numbers: JF323006 to JF323016.

**Number of amino acid residues: **amino acid residues were numbered according to the HA sequences of Qu/HK/G1/97 (H9) with GenBank accession number AF156378.

## Results

### Phylogenetic analyses of surface genes of H9N2 viruses

**Figure 1:**
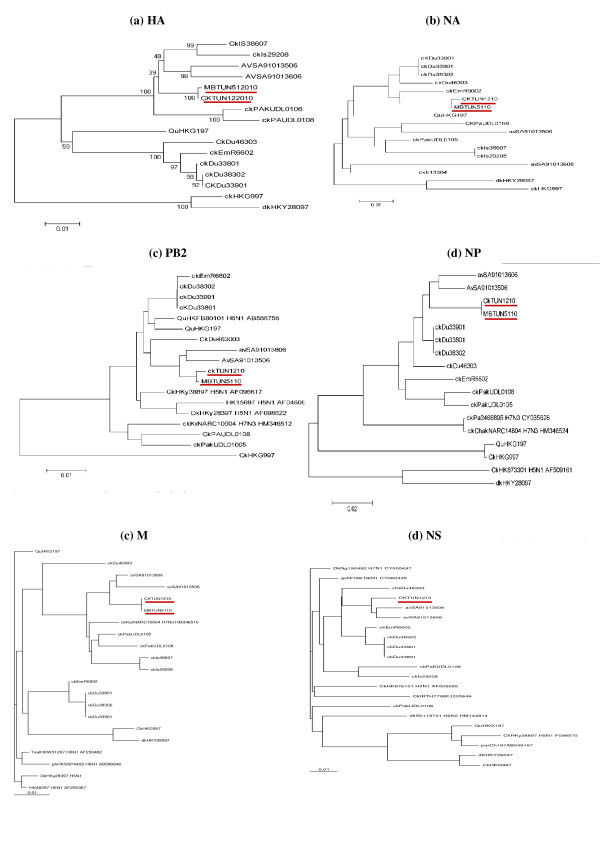
**Phylogenetic trees of the HA (a) (1072 bp from 184 to 1272), NA (b) (410 bp from 961 to 1370), PB2 (c) (540 bp from 1591 to 2130), NP (d) (326 bp from 1200 to 1529), M (e) (840 bp from 7 to 847) and NS (f) (890 bp from 1 to 890) genes of Tunisian Influenza A viruses**. The trees were generated by the distance-based neighbor-joining method using MEGA, version 5.0 program. The reliability of the trees was assessed by bootstrap analysis with 1, 000 replications; only bootstarp values of > 90% are shown. The length of the horizontal lines is proportional to the minimum number of nucleotide differences required to join nodes. The vertical lines are for spacing branches and labels. Abbreviations used in virus designation are as follows: Av, avian; Ck, chicken; Dk, duck; gs, goose; Qu, quail; Pa, paraket. The underlined strains are avian H9N2 Influenza viruses' isolates and sequenced in the present study.

To determine evolutionary relationships between Tunisian H9N2 isolates and those selected from the Genbank (Table 2), phylogenetic analyses were carried out for 6 viral gene segments (i.e., the genes for hemagglutinin (HA), neuraminidase (NA), polymerase (PB2), nucleoprotein (NP), matrix (M) and non structural (NS)) (Figure 1).

**Table 2:** Abbreviations used and GenBank accession numbers for H9N2 Avian Influenza virus isolates included in phylogenetic analysis

References strains	Abbreviations	Accession number					
		HA	NA	PB2	NP	M	NS
**Chicken/Tunisia/12/10**^**a**^	**Ck/TUN/12/10**	JF323006	JF323008	JF323010	JF323012	JF323014	JF323016
**Migratory bird/Tunisia/51/10**^**a**^	**MB/TUN/51/10**	JF323007	JF323007	JF323007	JF323007	JF323007	JF323007
Quail/Hong Kong/G1/97	QuHKG197	AF156378	AF156336	AF156435	AF156407	AF156463	-^b^
Duck/Hong Kong/Y280/97	DKHKY280097	AF156376	AF156394	AF156433	-	AF156461	AF156475
Chicken/Hong Kong/G9/97	CKHKG997	AF156373	AF156391	AF156430	AF156402	AF156458	AF156472
Chicken/Emirates/R66/02	CKEmR6602	CY076723	CY076725	CY076720	CY076724	CY076726	CY076727
Chicken/Dubai/338/01	CKDu33801	EF063520	EF063520	EF063555	EF063527	EF063506	EF063541
Chicken/Dubai/339/01	CKDu33901	EF063514	EF063521	EF063556	EF063528	EF063507	EF063542
Chicken/Dubai/383/02	CKDu38302	EF063515	EF063522	EF063557	EF063529	EF063508	EF063543
Chicken/Dubai/463/03	CKDu46303	EF063516	EF063523	EF063558	EF063530	EF063509	EF063544
Chicken/Pakistan/UDL-01/05	CKPaUDL0105	CY038458	CY038412	CY038407	CY038411	CY038461	CY038462
Chicken/Pakistan/UDL-01/08	CKPaUDL0108	CY038410	CY038460	CY038455	CY038459	CY038413	CY038462
Avian/Saudi Arabia/910135/06	AvSA91013506	GU050287	GU050297	GU050294	GU050287	GU050287	GU050287
Avian/Saudi Arabia/910136/06	AvSA91013606	GU050295	GU050289	GU050302	GU050298	GU050296	GU050287
Chicken/Israel/386/07	CkIs38607	-	FJ464619	-	FJ464636	FJ464602	FJ464653
Chicken/Israel/292/08	CkIs29208	FJ464617	FJ464616	-	-	FJ464599	FJ464650

^a ^Viruses whose HA, NA, PB2, NP, M and NS genes were sequenced in the present study; N.D, not done; ^b^, - No sequence data available.

Phylogenetic analyses based on the HA (Figure 1a) and NA (Figure 1b) genes (nucleotides 125 to 1272 and 961 to 1370, respectively), revealed that current Tunisian (H9N2) viruses are closely related to each other and shared closed relationship with the Middle Eastern strains, especially A/Av/SA/91013406/06, A/Ck/Em/R666/02, A/Ck/Du/338/01 and Ck/Pak/UL01/08. Percentage similarity scores, calculated from pairwaise alignments, supported the phylogenetic results and revealed a similarity score of more than 96% (Table 3). Tree analyses showed that Middle Eastern strains are grouped in G1 lineage and shared a common ancestor with A/Quail/Hong Kong/G1/1997 isolates.

**Table 3:** Percentage similarity between the gene segments of Tunisian isolates with those of reference viruses

**Identity (%)**^**a**^									
Tunisian isolates	Gene	AV/SA/06	CK/Em/R66/02	CK/Du/338/01	CK/Du/383/01	CK/Pak/UDL01/08	qu/HK/G1/97	qu/HK/Y280/97	Ck/HK/G9/97
	**HA**	**96.83**	92.45	92.91	92.73	**95.62**	93.66	88.81	89.65
	**NA**	92.00	**97.00**	**97.26**	**97.01**	94.00	95.00	93.53	92.53
**A/CK/TUN/12/10**	**PB2**	**95.72**	93.86	96.46	96.46	96.83	96.83	96.83	86.05
	**NP**	**98.2**	97. 30	**97.6**	**97.6**	92	85.87	85.5	90.71
	**M**	**98.46**	95.71	95.23	95.23	**97.18**	95.71	93	93.13
	**NS**	**97.77**	**96.53**	96.16	**96.61**	94.00	90.72	96.83	90.6
	**HA**	**96.74**	92.63	92.82	92.63	**95.52**	93.56	88.72	96.83
	**NA**	92.00	**97.26**	**97.26**	**97.26**	94.00	95.27	93.78	96.83
**A/MB/TUN/51/10**	**PB2**	**95.72**	93.86	96.46	**96.46**	92.00	85.87	85.50	96.83
	**NP**	**97.90**	97.70	**97.90**	**97.90**	94.91	97.30		96.83
	**M**	**98.46**	95.71	95.22	95.23	**97.18**	96.83	93.00	96.83

The highest similarities are in bold face

Virus abbreviation are listed in Table. 2

^a ^calculated based on the nucleotide sequences of HA, NA, PB2, NP, M and NS genes.

### Phylogenetic analyses of internal genes

Four internal genes (PB2, NP, M and NS) of Tunisian H9N2 strains showed more than 96% nucleotide identity with those of the Middle Eastern strains isolated from 2001 and 2008 (Table 3).

The phylogenetic studies of the PB2 genes were analysed using the 540- base-long nucleotide sequence (nucleotides 1591 to 2130) that codes the region of PB2 protein located between positions 530 and 710. Tree analysis of the PB2 gene showed that Ck/TUN/12/10 and Migratory bird/TUN/51/10 isolates are related to the Middle Eastern isolates (more than 97% similarity) and fall into a single group within the G1-like lineage (Figure 1c).

In the NP gene tree (Figure 1d), representative H9N2 viruses clustered into two distinct lineages, the G1-lineage including Middle Eastern, Qa/HK/G1/97, Asian strains, and Dk/HK/Y283/97 H5N1 like lineage. Besides, the H9N2 Tunisian isolates shared 97% to 98% similarity with other isolates such as A/Av/SA/91013406/06, A/Ck/Em/R666/02, A/Ck/Du/338/01 and showed a "sister group" relationship with H6N1 viruses of more than 97%.

The matrix genes (Figure 1e) (regions of the overlapping reading frames of M1-M2, nucleotides 7 to 845) of Ck/TUN/12/10 and MB/TUN/51/10 strains showed a close relationship to those of Middle Eastern (95% to 98% similarity), and to other subtypes such as, Ck/KHNC/100/04 H7N3 (96.93%), Dk/HK/Y283/97 H5N1 (95.74%), ph/HK/SSP44/02 H6N1 (95.23%) strains.

The NS gene (nucleotides 1591 to 2130) of Ck/TUN/12/10 showed evidence of reassortment with other viral subtypes. It was 95% and 92.9% similar to those of A/gs/11/99 (H6N1) and CK/HK/8761/01 (H5N1), respectively (Figure 1f).

### Molecular characteristics

To analyze the receptor binding site, the connecting peptide and the Hemadsorption site of neuraminidase specificity of the Tunisian isolates, we have compared the HA amino acid sequences (deduced from the nucleotide sequences) with those of representative viruses from GenBank.

### Hemagglutinin

The molecular determinants of pathogenecity and virulence of the HA protein are the HA1/HA2 connecting peptide sequence, specific amino acids (aa) residues at the receptor binding site (RBS), and the presence or absence of glycosylation sites around the receptor binding site.

HA cleavage site motif sequence of the Tunisian isolates was ^333 ^PARSSR* GLF^341 ^which is similar to other Asian H9N2 viruses including those from Hong Kong, Japan, Iran, Israel, Pakistan, and Saudi Arabia, Dubai. Residues at positions 110, 161, 163, 191, 198, 234, 235 and 236 are major components of the receptor binding site of the HA molecule. Tunisian viruses showed conservation of residues **P^110^, W^161^, T^163^, H^191^, A^198 ^**and **I^235 ^**in the receptor binding pocket. The left (aa residues at position 234-239) and right (aa residues at position 148-152) edge of the binding pocket motif were: NG**L/Q**IGR and GTSKS, respectively.

The A/CK/TUN/12/10 H9N2 strain carried the amino acid substitution (Q234L), a mutation that correlates with a shift in affinity of the HA from avian type sialic receptors to human type and from a preference for a **2^..^3^. ^**link to a **2^..^6^. ^**link between the sialic acid residues and galactose, whereas A/Migratory Bird/TUN/51/10 having Glu Q at this position. But, both strains had Gly (G) at position 228 at the RBS.

Analysis of HA protein sequences showed that Tunisian H9N2 isolates have many potential glycosylation sites with the N-X-T/S motif (X can be any amino acid expect proline): 82-84 (NPS), 105-107 (NGT), 141-143 (NVT), 218-220 (NRT), 298-300 (NST), 305-307 (NIS) (Table 4).

**Table 4:** Analysis of the amino acid sequences of the HA protein of Tunisian isolates with reference strains

			HA*								
	
**Virus**	**Receptor binding Site (RBS)**							**Left-edge of** **binding pocket**	**Right-edge of** **binding pocket**	**Connecting peptide** **aa sequence**	**Glycosylation site** **at position**
	**110**	**161**	**163**	**191**	**198**	**202**	**203**	**232**	**236 148**	**152 333**	**338 168**
	
**A/Ck/TUN/12/2010**	P	W	T	**H**	**A**	L	Y	N **G L I **G	G **T **S **K S**	P A **R **S S R	**+**
**A/M B/TUN/51/2010**	P	W	T	H	A	L	Y	N G **Q **I G	G **T **S **K S**	P A R S S R	**+**
**A/Av/SA/910136/06**	P	W	T	H	**T**	L	Y	N G L I G	G **T **S **K S**	P A R S S R	**-**
**A/Av/SA/910135/06**	P	W	T	H	A	L	Y	N G L I G	G **T **S **K S**	P A R S S R	**-**
**A/Ck/Du/338/01**	P	W	T	H	A	L	Y	N G L I G	G **T **S **K S**	P A R S S R	**-**
**A/Ck/Du/339/01**	P	W	T	H	A	L	Y	N G L **M **G	G **T **S **K A**	P A R S S R	**-**
**A/Ck/Du/383/02**	P	W	T	H	A	L	Y	N G L **M **G	G **T **S **K A**	P A R S S R	**-**
**A/Ck/Du/463/03**	P	W	T	H	A	L	Y	N G L **M **G	G **T **S **K A**	P A R S S R	**-**
**A/Ck/Em/R66/02**	P	W	T	H	A	L	Y	N G L **L **G	G **T **S **K A**	P A R S S R	**-**
**A/Ck/Is/386/07**	P	W	T	H	**E**	L	Y	N G **Q L **G	G **T **S **K S**	P A R S S R	**-**
**A/Ck/Is/292/08**	P	W	T	H	A	L	Y	N G L I G	G **T **S **K S**	P A R S S R	**+**
**A/Ck/Pak/UDL-01/0**	P	W	T	H	A	L	Y	N G L I G	G **T **S **K S**	P A R S S R	**+**
**A/Ck/Pak/UDL-01/08**	P	W	T	H	A	L	Y	N G L I G	G **T **S **K S**	P A R S S R	**-**
**A/Qu/HK/G1/97**	P	W	T	H	A	L	Y	N G L I G	G **T **S **K S**	P A R S S R	**-**
**A/DK/HK/Y280/97**	P	W	T	H	A	L	Y	N G L I G	G **T **S **K S**	P A **K **S S R	**-**
**A/Ck/HK/G9/97**	P	W	T	H	**E**	L	Y	N **D **L **Q **G	G **I **S **R A**	P A R S S R	**-**
	P	W	T	**N**	**T**	L	Y	N G L **Q **G	G **T **S **K A**	P A R S S R	**-**
	P	W	T	**N**	A	L	Y	N G L **Q **G	G **T **S **K A**	P A R S S R	**-**

*numbering according to H9, ^-/+ ^absence or presence of the Glycosylation site.

### HB site of Neuraminidase

Analysis of the neuraminidase active and framework sites of the NA protein revealed mutations in aa residues on 3 loops that interact directly with sialic acid; on loops carring amino acids 367-370-372, the three Ser (S) were substituted by KLA respectively, also D and Y substitution were found at residues 401, 406 respectively. Otherwise, the framework site contains R^371^, A^372^, N^402^, and E^425 ^(Table 5).

**Table 5:** Analysis of the amino acid sequences of the NA, PB2 and NP proteins of Tunisian isolates with reference strains

VIRUS	NA				PB2				NP					
	
	Neuraminidase active site(HB)	Framework site												
	366. 373	399. 406	431. 433	425	547	627	661	702	402	422	430	442	455	480
**A/Ck/TUN/12/2010**	IK**KDL**RAG	DSD**NW**SG**Y**	P**Q**E	E	**I**	E	**A**	K	**F**	R	K	T	D	D
**A/MB/TUN/51/2010**	IKKDLRAG	DSDNWSGY	PQE	E	I	E	A	K	**F**	R	K	T	D	D
**A/Av/SA/910136/06**	IK**EES**RAG	DSDN**L**SGY	PQE	E	I	E	A	K	S	R	K	T	D	D
**A/Av/SA/910135/06**	IKKDLRAG	DSDNWSGY	PQE	E	I	E	A	K	S	R	K	T	D	D
**A/Ck/Du/338/01**	IKKDLRAG	DSDNWSGY	PQE	E	**V**	E	A	K	S	R	K	T	D	D
**A/Ck/Du/339/01**	IKKDLRAG	DSDNWSGY	PQE	E	**V**	E	A	K	S	R	K	T	D	D
**A/Ck/Du/383/02**	IKKDLRAG	DSDNWSGY	PQE	E	**V**	E	A	K	S	R	K	T	D	D
**A/Ck/Du/463/03**	IKKDLRAG	DSDNWSGY	PQE	E	**V**	E	A	K	S	R	K	T	D	D
**A/Ck/Em/R66/02**	IKKDLRAG	DSDNWSGY	PQE	E	**V**	E	A	K	S	R	K	T	D	D
**A/Ck/Is/386/07**	IKKD**S**RAG	DSDNWSGY	PQE	E	**V**	E	A	K	S	R	K	T	D	D
**A/Ck/Is/292/08**	IKKD**S**RAG	DSDNWSGY	PQE	E	**V**	E	A	K	S	R	K	T	D	D
**A/Ck/Pak/UDL-01/05**	IKKD**S**RAG	DSDNWSGY	PQE	E	**V**	E	**V**	K	S	R	K	T	D	D
**A/Ck/Pak/UDL-01/08**	IKKD**S**RAG	DSDN**R**SGY	PQE	E	**V**	E	A	K	S	R	K	T	D	D
**A/Qu/HK/G1/97**	IKKD**S**RAG	DSDNWSGY	PQE	E	**V**	E	**T**	K	S	R	K	T	D	D
**A/DK/HK/Y280/97**	IK**E**D**S**RAG	DSD**IR**SG**S**	PQE	E	**V**	E	**V**	K	S	R	K	T	D	D
**A/Ck/HK/G9/97**	IKKD**S**RAG	DSDNWSGY	P**K**E	E	**V**	E	**T**	**K**	S	R	K	T	D	D
**A/Ck/HK/342/78(H5N1)**					**V**	E	A	K						
**A/Ck/HK/485/97(H5N1)**					**V**	E	A	K						
**A/Ck/Ch/NARC/148/04(H7N3)**					**V**	E	A	K	S	R	K	T	D	D
**A/Ck/Pa/34668/95(H7N3)**					**V**	E	A	K						
**A/Ck/HK/156/97(H5N1)**									**F**	R	K	T	D	D
**A/Dk/HK/Y283/97(H5N1)**									S	R	K	T	D	D
**A/Ck/HK/Y388/97(H5N1)**									S	R	K	T	D	D
**A/Qu/HK/FB801/01(H6N1)**									S	R	K	T	D	D
**A/Ck/Pa/34668/95(H7N3)**									S	R	K	T	D	D

### Molecular characteristics of internal proteins

The current Tunisian strains displayed Glu (E) at position 627 of the PB2 protein; therefore, a change to Lys could serve as a marker of the virulence phenotype. In addition, two avian virus-like aa at positions 661 (A) and 702 (K), similar to Ck/HK/8733/01 H5N1 strain isolated in 2001, were found (Table 5).

The nucleoprotein (NP) of Tunisian H9N2 strains retained conserved G1-lineage defining residues: R422, K430, T442, D455 and D480. However, Ser to Phe substitution at residue 402, found exclusively in Tunisian strains, similar to H5N1 strain circulated in 1997. Other aa specific for the nucleoprotein (NP) gene associated with host range, were not studied (Table 5).

Alignment of the M2 protein of current Tunisian H9N2 strains showed conserved G1 lineage defining Leu residue at position 10. None of the Tunisian isolates contained substitutions at amino acid positions 26, 27, 30, 31 or 34, suggesting the absence of resistance to M2 blockers antiviral drugs such as amantadine. However, we have identified some substitutions at position 16 (Gly, G), 28 (Ile, I), related to the ion channel domain and associated with host range. In addition, Ile, Val and Pro substitutions were present in Tunisian H9N2 viruses at residue 15 (V15I), 46 (L46V) and 54 (S54P) of M1 protein, respectively. Avian virus like aa such as: V^115^, T ^121 ^and T ^137 ^were also observed (Table 6).

**Table 6:** Analysis of the amino acid sequences of the NS and M proteins of Tunisian isolates with reference strains

VIRUS	NS1								NS2	M1			M2						
	
	Deletion of aa	Total of no of aa	PL motif									**NLS**^b^							
	80-84			92	103	106	184	217	71	15	46	54	16	26	27	28	30	31	34
**A/Ck/TUN/12/2010**	**NO**	**230**	GSEV	D	L	M	G	**N**	**S**	**I**	**V**	**P**	**G**	**L**	**V**	**I**	**A**	**S**	**G**
**A/MB/TUN/51/2010**	-	-	-	-	-	-	-	-	-	I	**V**	P	G	L	V	I	A	S	G
**A/Av/SA/910136/06**	NO	**230**	GSEV	**D**	L	**M**	G	**K**	**S**	I	**L**	**S**	**D**	L	V	**V**	A	S	G
**A/Av/SA/910135/06**	NO	**230**	GSEV	D	L	M	G	K	**S**	I	L	**S**	**D**	L	V	**V**	V	S	G
**A/Ck/Du/338/01**	NO	**220**	GSEV	D	**F**	M	G	K	**S**	I	L	P	G	L	**A**	V	A	S	G
**A/Ck/Du/339/01**	NO	**220**	GSEV	D	F	M	G	K	**S**	I	L	P	G	L	A	V	A	S	G
**A/Ck/Du/383/02**	NO	**220**	GSEV	D	F	M	G	K	**S**	I	L	P	G	L	A	V	A	S	G
**A/Ck/Du/463/03**	NO	**220**	GSE**I**	D	F	M	G	K	**S**	I	L	P	G	L	V	V	A	S	G
**A/Ck/Em/R66/02**	NO	**218**	GSEV	D	F	M	G	K	**S**	I	L	P	G	L	V	V	A	S	G
**A/Ck/Is/386/07**	NO	**230**	**K**SEV	D	F	M	G	K	**S**	I	L	**S**	G	L	V	V	A	S	G
**A/Ck/Is/292/08**	NO	**230**	GSEV	D	F	M	G	K	**S**	I	L	**S**	G	L	V	V	A	S	G
**A/Ck/Pak/UDL-01/05**	NO	**230**	GSEV	D	F	M	G	K	**S**	I	L	P	G	L	V	V	A	S	G
**A/Ck/Pak/UDL-01/08**	NO	**230**	**K**SE**I**	D	F	M	G	K	**S**	I	L	P	G	L	V	V	A	S	G
**A/Qu/HK/G1/97**	NO	**230**	**EP**EV	**E**	L	**I**	G	**N**	**S**	I	L	P	G	L	V	V	A	S	G
**A/DK/HK/Y280/97**	NO	**230**	**EP**EV	D	L	I	G	K	**S**	I	L	P	G	L	V	V	A	S	G
**A/Ck/HK/G9/97**	NO	**218**	**EP**EV	D	L	I	G	K	**S**	I	L	P	G	L	V	V	A	S	G
**A/Par/Ch/1/97**	NO	**230**	**EP**EV	**E**	L	I	G	**N**	**S**	I	L	P	G	L	V	V	A	S	G
**A/DK/HK/Y388/97(H5N1)**	NO	**218**	**EP**EV	**E**	L	I	G	**N**	**S**	I	L	P	G	L	V	V	A	S	G
**A/DK/HK/8761/01(H5N1)**	**YES**	**230**	**E**SEV	D	F	M	G	**N**	**S**	I	L	P	G	L	V	V	A	S	G
**A/Ck/Ng/1904/92(H7N1)**	NO	**230**	**E**SEV	D	F	M	G	**N**	**S**	I	L	P	G	L	V	V	A	S	G
**A/gs/NL1/99(H6N1)**	NO	**230**	**E**SEV	D	F	M	G	**N**	**S**	I	L	P	G	L	V	V	A	S	G
**A/Ck/Sh/1197/01(H6N1)**	NO	**230**	**E**SEV	D	F	M	G	**N**	**S**	I	L	P	G	L	V	V	A	S	G

^a ^PL motif is referred as PDZ-binding motif at the C-terminal end of the NS1 protein, ^b ^NLS is referred to nuclear localization signal, ^- ^not done.

Based on the analysis of the non structural protein gene (NS), our recent isolates did not show the five amino acid (80TIAS84) deletions, resulting in an NS1 protein of 230 amino acid in length and containing the "GSEV" PDZ ligand (PL) C-terminal motif, and did not harbor the mutation of Asp (D) to Glu (E) at position 92, required for high virulence. Besides, The RNA-binding domain exhibited amino acid residues P31, D34, R35, R38, K41, G45, R46 and T49. However, the effector domain carried the F103L, R184G and K217N substitutions. Moreover, E227G mutation in C-terminal domain of the NS1 protein, introducing an S70I mutation into nuclear export protein (NEP), was also observed (Table 6).

## Discussion

Infections of domestic poultry, especially chickens with H9N2 subtype of avian influenza virus (AIV) have been frequently reported in China, other Asian and North American countries, since the late 1990s. They have been grouped in different sublineages on the basis of antigenic and genetic properties. Although, H9N2 viruses do not satisfy the criteria for highly pathogenic avian influenza, they are unique among this category, infecting a wide variety of species, including chickens [21,22], quails [23], pigeons [24], turkeys [25], ducks [25-27], geese, pigs [28], and humans. Several studies have reported that G1- like H9N2 viruses can infect humans and can replicate in human alveolar epithelial cells and mouse respiratory system. Interestingly, they could emerge as human pathogens though reassortement in intermediate hosts, such as pigs and avian species, or through direct adaptation to human.

Since late 2009, H9N2 influenza A viruses have caused many outbreaks in Tunisian flocks. A second wave of AI was reported during July-October 2010. Here, we have provided the first comprehensive genetic data for the H9N2 subtype viruses circulating in Tunisian poultry flocks. Strains were collected from two different governorates situated in the northeastern part of Tunisia. Our findings revealed that H9N2 virus infection is well established in some Tunisian endemic areas. Therefore, understanding the genetic and the biological characteristics of H9N2 virus isolated from different species and regions can provide a comprehensive insight into the biology of H9N2, the ecology of AI virus, and the ability of migratory birds to disseminate influenza viruses.

The Blast analysis (NCBI) of the nucleotide sequences of HA and NA genes showed that A/Ck/TUN/12/10 and A/Migratory Bird/TUN/51/10 were the most closely related to the Middle Eastern isolates belonging to the G1- like lineage in the H9N2 subtype (more than 96.5% similarity).

The results of the phylogenetic analyses were basically in agreement with the blast data, and confirmed that our isolates fall, together with the Middle Eastern strains, into a distinct cluster, related to the G1 lineage; a finding that may indicate a common origin.

The internal PB2, NP, M, NS genes were also similar to those of other Middle Eastern strains, which can be traced back to the same G1- like lineage. In addition, NP and NS genes seemed to have undergone broad reassortments with H5N1, H7N3, H7N1, H6N1 influenza virus subtypes as described for previous isolated strains [18,29,30]. This diversity reflects an increased spread of such viruses through avian species' migration. It is difficult to explain whether and how these reassortments have occurred because of the lack of local and regional epidemiological information.

Based on the deduced amino acid sequences, the HA1-HA2 connecting peptides of the Tunisian avian H9N2 isolates did not harbor multiple basic amino acids: PARSSR/GL as found for recently isolated H9 viruses in Middle East [31] and Asia [32,33]. This might indicate the LPAI nature of H9N2 strains, although the motif for these viruses is similar to the RX-RYK-R required for the highly pathogenic H5 and H7 subtypes [34,35]. These genetic findings suggested that our H9N2 viruses may have the potential to acquire basic amino acids in the HA connecting peptide sequence needed to become highly pathogenic through the addition of single basic amino acid at the -4 position.

Moreover, A/Migratory bird/TUN/51/10 possessed a Glu (Q) at position 234 in the HA1 portion (H9 numbering; 226 in H3 numbering), whereas A/Ck/TUN/12/10 showed a leu (L), a receptor binding site residue, typical for human influenza virus displaying human virus-like cell tropism through an association with a preferential binding of sialic acid (SA) to galactose in α 2, 6 linkage [9,36]. A leu (L) residue at position 234 in the HA receptor binding site (RBS) was found to be important for the transmission of the H9N2 viruses in ferrets [37]. In previous study, Wan suggested that Q234L substitution, found in G1, Y280 and G9 lineages, isolated in Hong Kong, allows H9N2 viruses to preferentially infect non ciliated cells and grow more efficiently in human airway epithelial cell cultures; thus, increasing the infection severity in humans [12]. Our findings suggested that A/Ck/TUN/12/10 may have segregated from the migratory A/Migratory bird/TUN/51/10 strain acquiring an affinity for the human receptor binding profile. These findings may be attributed to a particular introduction of a new virus that has been circulating within poultry species.

Remarkably, residue at position 198 within the receptor binding pocket has been reported to influence the affinity of virus binding to SA receptor; high affinity to the human like receptor being with V at position 198, intermediate with T and low with A [38]. It can be predicted that the Tunisian A/Ck/TUN/12/10 isolate has a weaker affinity binding to human like receptor, but this finding need to be further confirmed by experimental studies.

Analysis of potential glycosylation site motif N-X-S/T in the HA1 protein of Tunisian H9N2 isolates, revealed some sites at positions 82, 105, 141, 298, 305. Additional glycosylation site at position 168 was observed compared with representative reference strains. It has been suggested that the alteration in the glycosylation pattern influences the adaptation of avian influenza viruses to poultry by altering their pathogenecity and antigenicity and helps to the evasion from the host antibody response [39-41].

The NA of the new Tunisian isolates carried substitutions in the HB site, similar to those of other avian H9N2 viruses isolated in Asia and Middle Eastern during human pandemic H2N2 and H3N2 that bind to α -2, 6 -linked receptors [42]. These viruses were showed to be under a positive selection pressure, resulting in compatible combinations of HA and NA [38].

Based on the deduced amino acid sequence, the Tunisian H9N2 isolates displayed Glu (E) at position 627 of the PB2 protein. Likewise, a single aa substitution in PB2 protein (E627K) can dramatically alter the virulence and enhance viral replication in mice [43] and other mammals [44-46]. Two avian virus-like aa at positions 661 (A) and 702 (K), as seen in H5N1 strains, were identified in the PB2 protein. These specific aa are located in the functional domain responsible for interaction with other polymerase components [47].

The main function of the NP is encapsidation of the viral genome to form a ribonucleoprotein particle for transcription and packaging; it interacts with other viral PB1, PB2, M1 and cellular proteins (Importin α, F-actin, CRM1/exportin 1) for viral transcription and nuclear transportation controls [48]. The NP aa sequence of the Tunisian isolates retained conserved G1-lineage defining residues. Interestingly, a new mutation (S402F), which has not been seen before, was detected in the NP protein. This mutation was previously found in the H5N1 subtype instead of Ser in all other H9N2 subtypes compared in this study (Table 5).

It has been reported that aa residues at positions 15, 115, 121, 137, 240 in virus matrix protein are linked with increased replication in mammals or increased pathogenecity in small animal models [49,50]; an Ile substitution was present in Tunisian H9N2 viruses at aa position 15 (V15I). No one contained substitutions at aa positions 26, 27, 30, 31 or 34 within the transmembrane domain of M2 protein, maintaining a genotype associated with sensitivity to adamantadine M2 blocker antiviral drugs [51]. It has been known that amantadine binds to the ion channel region of the M2 protein and prevents the release of viral RNA into cells [52]. Tunisian H9N2 strains harbor two human virus- like amino acids at positions 16 (Gly, G) and 28 (Ile, I) which are related to the ion channel domain and associated with host range (49, 50), but the role of these substitutions on increased replication efficiency in mammalian cells is not yet known.

A Val substitution at position 46 was also detected for the first time in our strains; however, all other H9N2 and other IA subtypes have leu at this position (Table 6). Interestingly, this substitution should be considered.

Recent studies have suggested that NS1 protein suppresses the host antiviral defenses at multiples levels and correlation between NS1 and virulence has been reported [53-55]. Molecular analysis showed that our isolates contained an NS1 protein, with 230 aa in lengh, typical of H9N2 viruses. A recent study has shown that increasing the length of the 2009 H1N1 NS1 protein to 230 aa does not increase virus replication in human and pig cells [56]. Other study showed that viruses containing NS1 truncations were found to induce more interferon than viruses with full- length NS1 proteins and were correspondingly more attenuated in mice [57].

In the RNA- binding domain of NS1, A/Ck/TUN/12/2010 isolate contained R38 and K41, which are shown to be critical for RNA binding. as well amino acid residues P31, D34, R35, G45, R46, T49 and D55, which also mediate NS1-dsRNA interaction. Residue 55 is located within the third alpha- helix (residues 54-70) of the dsRNA-binding domain (RBD (residues 1-73) of NS1 [58]. It is documented that variation of NS1-55 from Asp (D) to gly (G) represents loss of changed aa which may stabilize the coiled- coiled helical structure. In addition, our strains exhibited no differences in the second nuclear localization sequences (NLS2) motif, or change in the amino acid D at position 92. Some studies reported a mutation of asp (D) to Glu (E) to be related to virulence of H5N1 in mammalian species and cytokine resistance [59]. Nevertheless, their effector domain carried Leu at position 103 and Gly at residue 184. In fact, F103L and M106I mutations are adaptive genetic determinants of growth and virulence in both human and avian NS1 genes in the mouse model [60]. Likewise, it has been demonstrated that, in addition to its contribution to cleavage and polyadenylation specificity factor (CPSF) binding, Gly 184 strongly influence viral virulence by an unknown mechanism which does not involve the INF system [61].

An Asp (N) was found at residue 217 only in Ck/TUN/12/2010 strain that differs from the other H9N2 strains which have K at this position, but similar to those of H5N1 strain CK/HK/8761/01 (Table 4). However, the biological significance of this substitution is not yet known.

Also, Ck/TUN/12/2010 didn't exhibit a five amino (80TIAS84) deletion observed in 2001 in poultry in Hong Kong and has, since, became the most common sequence found in the HP viruses. Long et al, demonstrated that viruses containing NS1 with 5 amino acid deletion (80TIAS84) residues showed increased virulence in both mouse and poultry [62] but the biological significance of these deletions is not fully understood yet.

Fortunately, previous study, performed on large scale sequence analysis of viruses isolated from different birds and mammalian species, have identified that the C-terminal domain of NS1 functions as a species-specific virulence domain: the vast majority of avian influenza viruses have an NS1 protein with a PDZ ligand (PL) C-terminal ESEV domain, while typical human viruses have a conserved RSKV domain. NS1 proteins with C-terminal ESEV, KSEV, and EPEV domains were shown to bind to PDZ domains containing cellular proteins [63,64]. Soubies et al demonstrated that RSKV motif, which lacks a PDZ- binding domain, replicated to higher titers than ESEV in humans and ducks cells, suggesting the ability of NS1 to interact with PDZ containing proteins does not contribute to virulence in the host species [65]. Nevertheless, it has been showed that insertion of four C-terminal aa, either ESEV, KSEV, or EPEV, into avirulent viruses resulted in an increase in virus virulence and caused severe disease in mice [52]. The H9N2 viruses recently isolated in Tunisia, have a PL motif "GSEV" previously found in Dubai strains during 2001-2003 (data not published); but the biological signification of this motif is unknown. Interestingly, the E227G mutation in NS1 introduces an S70I mutation into nuclear export protein.

## Conclusion

In the present study, we characterized two Tunisian H9N2 strains isolated from disease outbreaks in commercial chicken and migratory bird from different geographic areas. Our findings indicate that CK/TUN/12/10 isolate has evolved and reassorted with other influenza viruses and has acquired an affinity for the human receptor binding profile. Otherwise, the biological significance of new detected substitutions founded in some internal genes and the PDZ ligand polymorphism of the NS protein should be tested.

Actually and face to the high mortality, we are preparing for an active program to develop and evaluate vaccines against H9N2 to control the further spread of the disease in the industrial poultry production sectors. Interestingly, continuous monitoring of viral genetic changes throughout the years is warranted to monitor variations of Influenza viruses in the field.

## List of abbreviations

aa: amino acid; AIV: Avian Influenza virus; HA: hemagglutinin; HB: heamadsorption; HPAIV: highly pathogenic type; LPAI: Low pathogenic type; M: matrix; NA: neuraminidase; NP: nucleoprotein; NS: non structural; PB2: polymerase; PL: PDZ ligand; RBS: receptor binding site

## Competing interests

The authors declare that they have no competing interests.

## Authors' contributions

WT is a PhD, who carried out the H9N2 avian influenza virus genes detection by RT-PCR and was involved in the sequencing assays, phylogenetic and amino acid analysis, coordinated the work described, and drafted of the manuscript. JN and IL were involved in its design and coordination. JLG involved in drafting the manuscript or revising it critically for important intellectual content. AG conceptualized the study, supervised all facets of the research, and involved in design of the trials, assisting in the writing process. All authors read and approved the final manuscript.
